# Clinical Decision-Making Regarding the Added Value of Postoperative Chemotherapy After Complete Secondary Cytoreductive Surgery in Unifocal First Recurrent Epithelial Ovarian Cancer—An International Expert Survey

**DOI:** 10.3390/jpm16090449

**Published:** 2026-08-28

**Authors:** Renée E. W. M. van de Vorst, Sophie S. M. Pelk, Eelke H. Gort, Petronella O. Witteveen, Ronald P. Zweemer, Cornelis G. Gerestein

**Affiliations:** 1 Department of Gynecological Oncology, University Medical Center Utrecht, 3584 CX Utrecht, The Netherlandsc.g.gerestein-2@umcutrecht.nl (C.G.G.); 2 Department of Medical Oncology, University Medical Center Utrecht, 3584 CX Utrecht, The Netherlands

**Keywords:** epithelial ovarian cancer, secondary cytoreductive surgery, postoperative chemotherapy, unifocal recurrence, recurrent ovarian cancer, expert survey

## Abstract

**Background**: Secondary cytoreductive surgery (SCS) followed by postoperative chemotherapy is a recognized option for select patients with platinum-sensitive first recurrent epithelial ovarian cancer (EOC), supported by the DESKTOP-III and SOC-1 trials. Neither trial reported chemotherapy rates stratified by lesion number or resection status. As a result, the added value of postoperative chemotherapy after complete resection of a unifocal recurrence remains unclear. This study aimed to evaluate current clinical practice and factors influencing this decision. **Methods**: A cross-sectional survey was conducted among gynecological and medical oncologists from international expert oncological centers, assessing definitions of unifocal recurrence, current postoperative chemotherapy practice after complete SCS, perceived clinical value and strength of the supporting evidence, and factors influencing decision-making. **Results**: Twenty of 48 invited experts responded (41.7%). Respondents applied different definitions of unifocal recurrence, leading to variable estimates of the annual number of patients undergoing SCS. Respondents perceived postoperative chemotherapy to have meaningful clinical value, despite rating the supporting evidence as weak. A proportion did not routinely administer postoperative chemotherapy after complete SCS, managing patients with observation alone. Decisions to omit chemotherapy were influenced by institutional, clinical, and patient-related factors. Overall, 82% of respondents considered a randomized trial comparing chemotherapy with observation clinically valuable. **Conclusions**: Clinical practice regarding postoperative chemotherapy after complete SCS for a first unifocal recurrence of EOC varies among international expert oncological centers. The absence of a uniform definition of unifocal recurrence and lack of direct evidence regarding postoperative chemotherapy omission highlight the need for standardized definitions and prospective randomized evaluation of chemotherapy versus observation.

## 1. Introduction

Secondary cytoreductive surgery (SCS) has increasingly been incorporated into the treatment strategy for selected patients with platinum-sensitive first recurrent epithelial ovarian cancer (EOC). Randomized trials have demonstrated improved survival outcomes when patients are randomized to SCS in combination with postoperative chemotherapy compared to chemotherapy alone, with the most pronounced survival benefits observed in patients achieving complete macroscopic resection [1,2,3,4,5,6,7].

In the DESKTOP-III trial, patient selection was based on the Arbeitsgemeinschaft Gynäkologische Onkologie score [1,2,3]. Patients treated with SCS followed by postoperative chemotherapy experienced significant improvements in progression-free survival (18.4 vs. 14.0 months) and overall survival (53.7 vs. 46.0 months) compared to chemotherapy alone. Complete macroscopic resection was achieved in 75.5% of patients and was associated with the most pronounced survival benefit, with a median overall survival of 61.9 months. Postoperative chemotherapy was administered to most patients, with 76.7% of patients in the surgery group and 79.6% in the chemotherapy-alone group completing at least five cycles of platinum-based second-line therapy.

The SOC-1 trial provided complementary evidence using selection based on the iMODEL score combined with PET–CT imaging [6,7]. SCS followed by chemotherapy resulted in a significant progression-free survival benefit (17.4 vs. 11.9 months; HR 0.58). Median overall survival was 58.1 months compared with 52.1 months with chemotherapy alone (HR 0.80, 95% CI 0.61–1.05). After adjustment for crossover of 35% from chemotherapy alone to SCS, a significant overall survival benefit was observed (adjusted HR 0.76, 95% CI 0.58–0.99). Postoperative chemotherapy was administered to nearly all patients, with 96.7% in the surgery group and 96.0% in the control group receiving an assigned platinum-based regimen.

Within the SOC-1 trial, complete macroscopic resection was achieved in 77% of patients undergoing SCS and was associated with a median overall survival of 73.0 months. Prespecified subgroup analyses according to tumor lesions demonstrated a clear association between tumor lesion number and overall survival. In patients with 1–3 lesions, complete macroscopic resection was achieved in 93% and median overall survival was 102.6 months. Among patients with 4–19 lesions, complete resection was achieved in 83%, with a median overall survival of 69.5 months. In contrast, patients with ≥20 lesions experienced poorer survival, with a median overall survival of 36.3 months.

These data indicate that achieving complete macroscopic resection represents the strongest prognostic factor after SCS. Neither DESKTOP-III nor SOC-1 reported whether the proportion of patients receiving postoperative chemotherapy varied according to tumor lesion number or complete resection status, despite high overall chemotherapy administration rates in both trials. From a clinical perspective, unifocal recurrence is generally considered more surgically accessible and has been associated with higher rates of complete macroscopic resection compared with multifocal disease [1,2,6,8,9]. However, macroscopically complete resection does not exclude the presence of microscopic residual disease, and postoperative chemotherapy may still eradicate occult metastatic disease even after apparently complete surgical resection. This raises the question of whether postoperative chemotherapy could potentially be omitted in patients with unifocal recurrence achieving complete resection, which formed the rationale for the present study.

In our recent systematic review, authored by Van de Vorst et al., we evaluated treatment outcomes after SCS in patients with unifocal versus multifocal recurrence, with unifocal recurrence defined as one to two lesions in the included studies [10]. Unifocal recurrence was consistently associated with longer progression-free and overall survival compared with multifocal recurrence when treated with SCS followed by chemotherapy. However, no studies directly compared SCS alone with SCS followed by chemotherapy in patients with completely resected unifocal recurrence, leaving the added value of postoperative chemotherapy in this subgroup undefined.

Current ESGO–ESMO recommendations recommend SCS followed by postoperative chemotherapy in selected patients with platinum-sensitive first recurrent EOC, whereas the Society of Gynecologic Oncology emphasizes the heterogeneity of the evidence and advises cautious interpretation when considering this approach [11,12,13]. Reflecting the lack of subgroup-specific evidence, these recommendations do not specifically address patients with completely resectable unifocal first recurrence, resulting in ongoing clinical uncertainty. Therefore, we conducted an international survey among clinicians working in expert oncological centers to (1) characterize current expert practice regarding postoperative chemotherapy after complete SCS for unifocal first recurrence of EOC, and (2) identify factors influencing treatment decisions.

## 2. Materials and Methods

### 2.1. Study Design

A cross-sectional expert survey study was conducted among experts from centers worldwide, defined as practicing gynecological and medical oncologists with demonstrated clinical or academic involvement in the management of recurrent EOC. Experts were identified through direct outreach to members of the ESGO–ESMO Guidelines Committee, as well as to the principal investigators of the national, multicenter DUTCH PROSPECT study in the Netherlands, which focuses on this specific patient population [12,13]. A systematic literature search was conducted to identify additional experts with last authorship of peer-reviewed publications on SCS and postoperative chemotherapy in recurrent EOC. The methodology of this literature search has been described previously and is included in Appendix A [10,12,13]. A total of 48 experts in this field were identified worldwide. This survey study was submitted to the institutional review board and was assessed as exempt from ethical approval in accordance with the Medical Research Involving Human Subjects Act (26U-0081).

### 2.2. Procedure

Eligible gynecological oncologists and medical oncologists were contacted by e-mail between February 2026 and June 2026 and received a personalized link to the survey. The survey was developed and distributed via Castor, a clinical data management platform [14]. The survey was conducted in English. Clinicians who did not complete the survey received up to four reminder emails, followed by telephone follow-up for persistent non-responders. Participation was voluntary, and no incentives or compensation were provided.

### 2.3. Survey

The survey was newly structured according to the 2022 Consolidated Framework for Implementation Research (CFIR) [15]. The survey included questions assessing experts’ baseline characteristics and clinical experience, as well as surgical indications, the definition of unifocal recurrence, and the clinical value, current evidence, and decision-making factors related to postoperative chemotherapy following SCS. The original questionnaire is provided in Appendix B. The survey included a range of question formats, including binary and multiple-choice questions, multi-select items, 5- and 10-point Likert scales and open-ended responses. The survey questions were reviewed by five clinicians (RV, EG, PW, RZ and CG).

### 2.4. Data Analyses

Data was analyzed using R version 4.5.2 [16]. Descriptive statistics were calculated based on the number of responses per item. Continuous variables, including slider-scale responses, were presented as means with standard deviations or medians with interquartile ranges, as appropriate. Categorical variables were summarized using frequencies and proportions. Open-ended responses were reviewed descriptively and used to contextualize quantitative findings.

## 3. Results

### 3.1. Respondents Characteristics

A total of 48 experts from 44 centers worldwide were invited to participate in the survey. Twenty responses were received, resulting in a response rate of 41.7% (Table 1). Respondents represented 20 centers from seven countries: Germany (*n* = 1), Italy (*n* = 1), the Netherlands (*n* = 13), Norway (*n* = 1), Spain (*n* = 1), Turkey (*n* = 1) and United Kingdom (*n* = 2). Almost all respondents had a primary field of expertise in gynecological oncology (*n* = 18/20; 90%), and the majority work at a tertiary center (*n* = 14/20; 70%) and have more than 10 years of practice experience (*n* = 17/20; 85%). Three questionnaires were returned incompletely and were included for the answered items, as specified per analysis. Incompleteness was due to missing responses, unless stated otherwise.

### 3.2. Surgical Volume and Definition of Unifocal Recurrence

According to their local definition of unifocal recurrence, 19 respondents reported a median of 5 patients per year (range 0–30) undergoing SCS for a first unifocal recurrence of EOC at their center. When asked to define unifocal recurrence, 11 respondents (*n* = 11/19; 58%) restricted the definition to a single lesion, while one respondent (*n* = 1/19; 5%) accepted up to two lesions and seven respondents (*n* = 7/19; 37%) up to three lesions (Table 2). Anatomical location was considered relevant by 14 respondents (*n* = 14/20, 70%), with multiple answers allowed: 4 respondents (*n* = 4/14; 29%) considered multiple lesions within a single organ to be unifocal, 5 (*n* = 5/14; 36%) within a single anatomical compartment, and 6 (*n* = 6/14; 43%) within a single lymph node region.

Several respondents added nuance in free text. One respondent noted that their center uses the term locoregional rather than unifocal recurrence, and another respondent distinguished unifocal recurrence (a single site) from oligometastatic disease (up to five sites) in line with ESMO guidelines. Two respondents considered the anatomical distribution of lesions and the feasibility of achieving complete cytoreduction to be more important than the absolute number of lesions. One respondent considered disease confined below the diaphragm to be compatible with unifocal recurrence. For comparability, respondents were asked to apply a standardized definition of unifocal recurrence—a single recurrent lesion irrespective of anatomical location—even where this differed from their own clinical practice. Under this definition, 18 respondents reported a median of 3.5 patient per year (range 0–30) undergoing secondary cytoreductive surgeries for unifocal first recurrence of EOC at their center.

### 3.3. Clinical Added Value, Strength of Current Evidence and Guideline Influence

**Figure 1:**
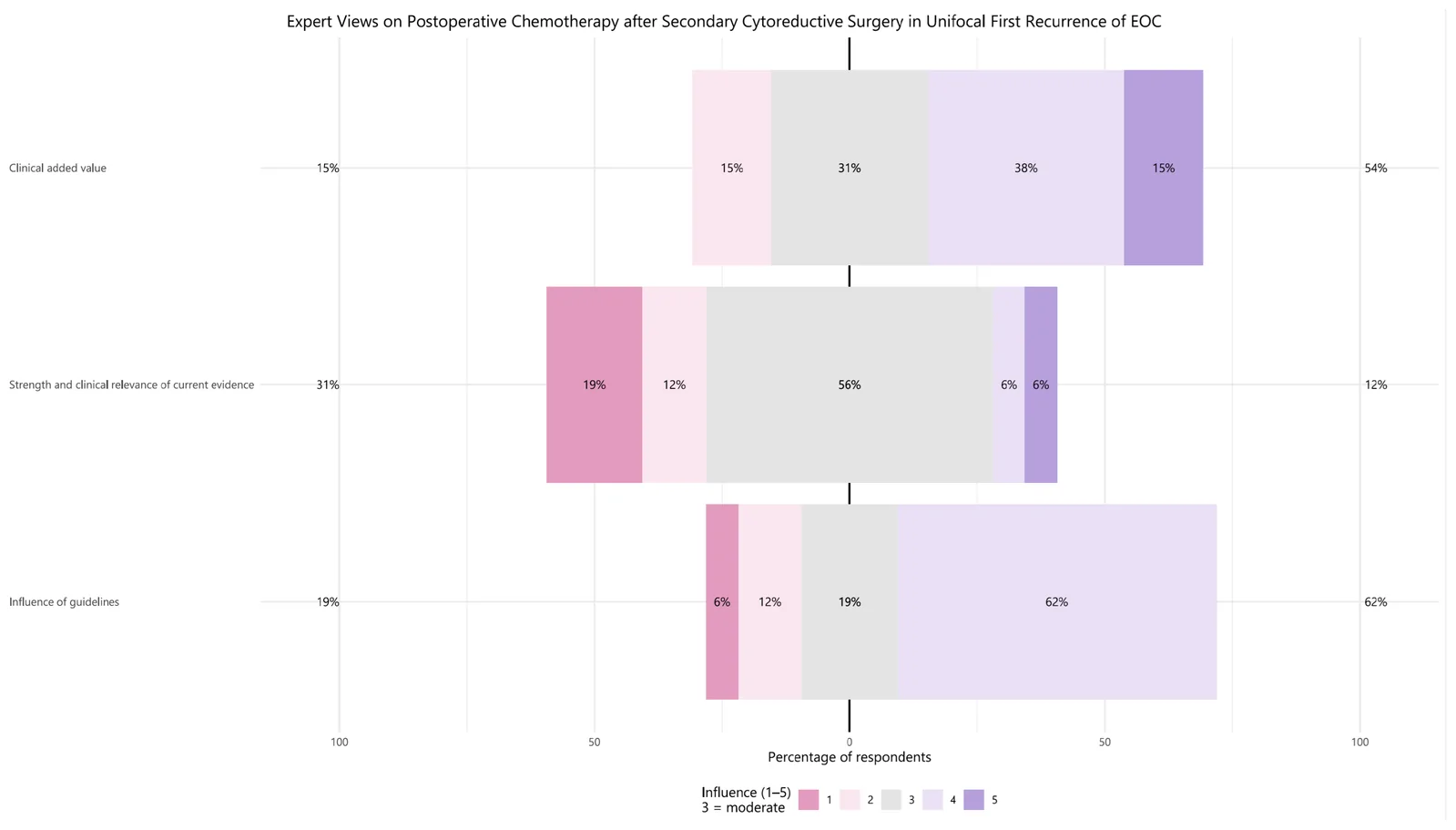
Expert Views on Postoperative Chemotherapy after Secondary Cytoreductive Surgery in Unifocal First Recurrence of EOC (*n* = 17). Ratings of the perceived clinical added value, strength and clinical relevance of current evidence, and the influence of (inter)national guidelines on the recommendation of postoperative chemotherapy after secondary cytoreductive surgery in unifocal first recurrence of EOC. Each on a 5-point Likert scale (1 = lowest, 3 = moderate, 5 = highest). Unsure responses were excluded. Percentages may not sum to 100% due to rounding.

Respondents rated clinical added value, strength of current evidence and national or international guideline influence on postoperative chemotherapy after SCS in unifocal first recurrence of EOC on a 5-point Likert scale (*n* = 17/20) (Figure 1). Unsure responses were most frequent for added clinical value (*n* = 4/17; 24%) and less so for the strength of evidence and the influence of guidelines (*n* = 1/17, 6% each) and were excluded from the distributions below. Among the 13 evaluable respondents, the clinical added value of postoperative chemotherapy after SCS was rated as moderate to significant by 54% (*n* = 7/13), whereas 15% (*n* = 2/13) rated it as limited to no added value. The strength of the supporting evidence was rated as moderate or strong by 12% (*n* = 2/16), with 31% (*n* = 5/16) considering it limited to insufficient. National or international guidelines were reported to be very influential or to completely determine the recommendation by 62% (*n* = 10/16).

### 3.4. Adherence to the DESKTOP-III and Use of Observation Only

When asked whether all patients who have undergone complete SCS receive postoperative chemotherapy, as applied in the DESKTOP-III trial, 8 respondents (*n* = 8/17; 47%) reported that all patients are treated this way, 8 (*n* = 8/17; 47%) that most patients are, and 1 (*n* = 1/17; 6%) that postoperative chemotherapy is not routinely given. Seventeen respondents reported managing a median of 1 patient per year (range 0–10) with observation alone after complete SCS at their center.

### 3.5. Clinician- and Patient-Related Factors Influencing the Postoperative Chemotherapy Decision After Complete Secondary Cytoreductive Surgery

**Figure 2:**
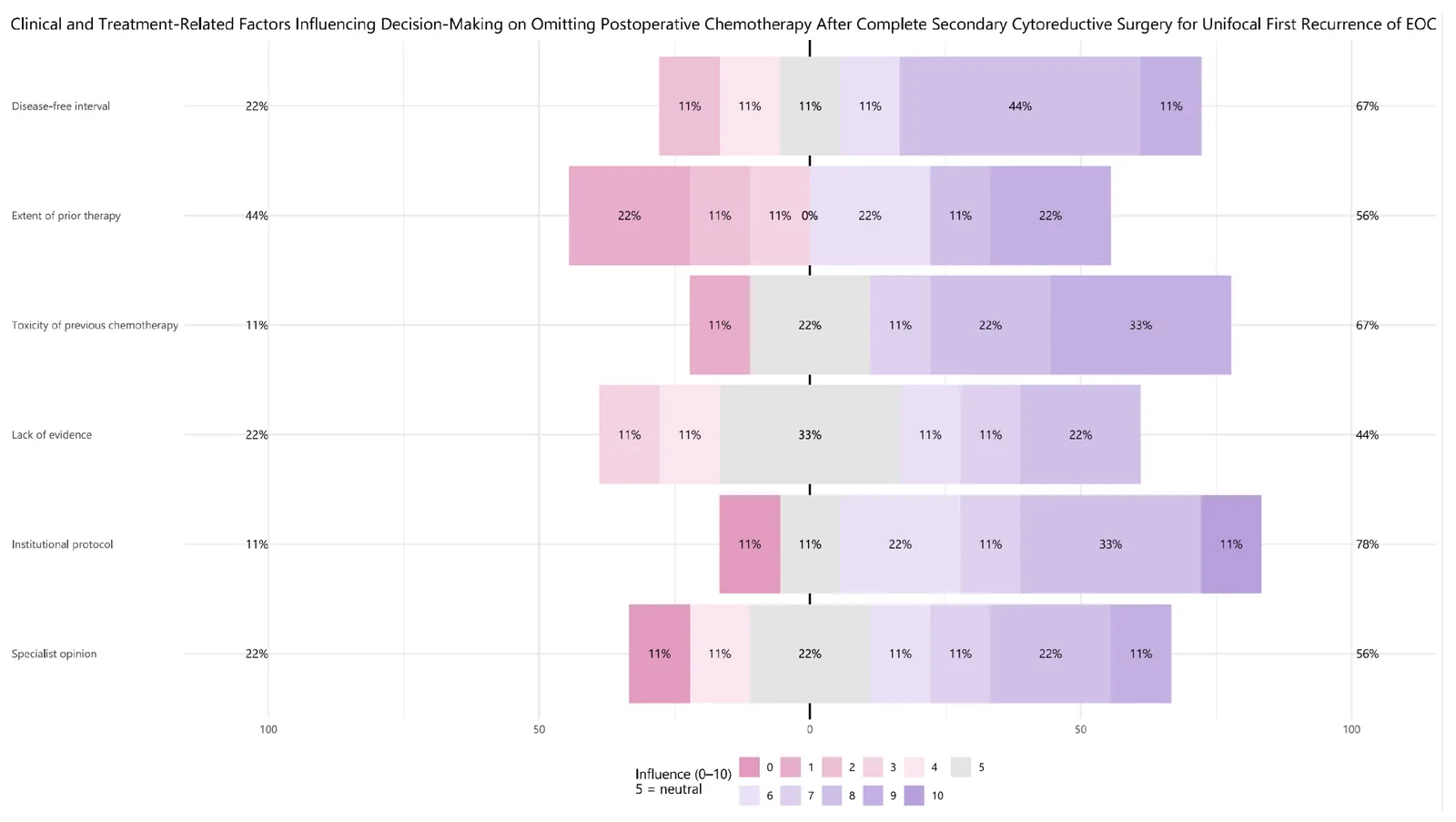
Clinician- and treatment-related factors influencing decision-making on omitting postoperative chemotherapy after complete secondary cytoreductive surgery in unifocal first recurrence of EOC (*n* = 9). Ratings of the influence of the disease-free interval, the extent of prior systemic therapy, toxicity from previous chemotherapy, the lack of high-level evidence, institutional protocol/local consensus, and the opinion of a key specialist, each on a 0–10 scale of influence (5 = neutral). Percentages may not sum to 100% due to rounding.

**Figure 3:**
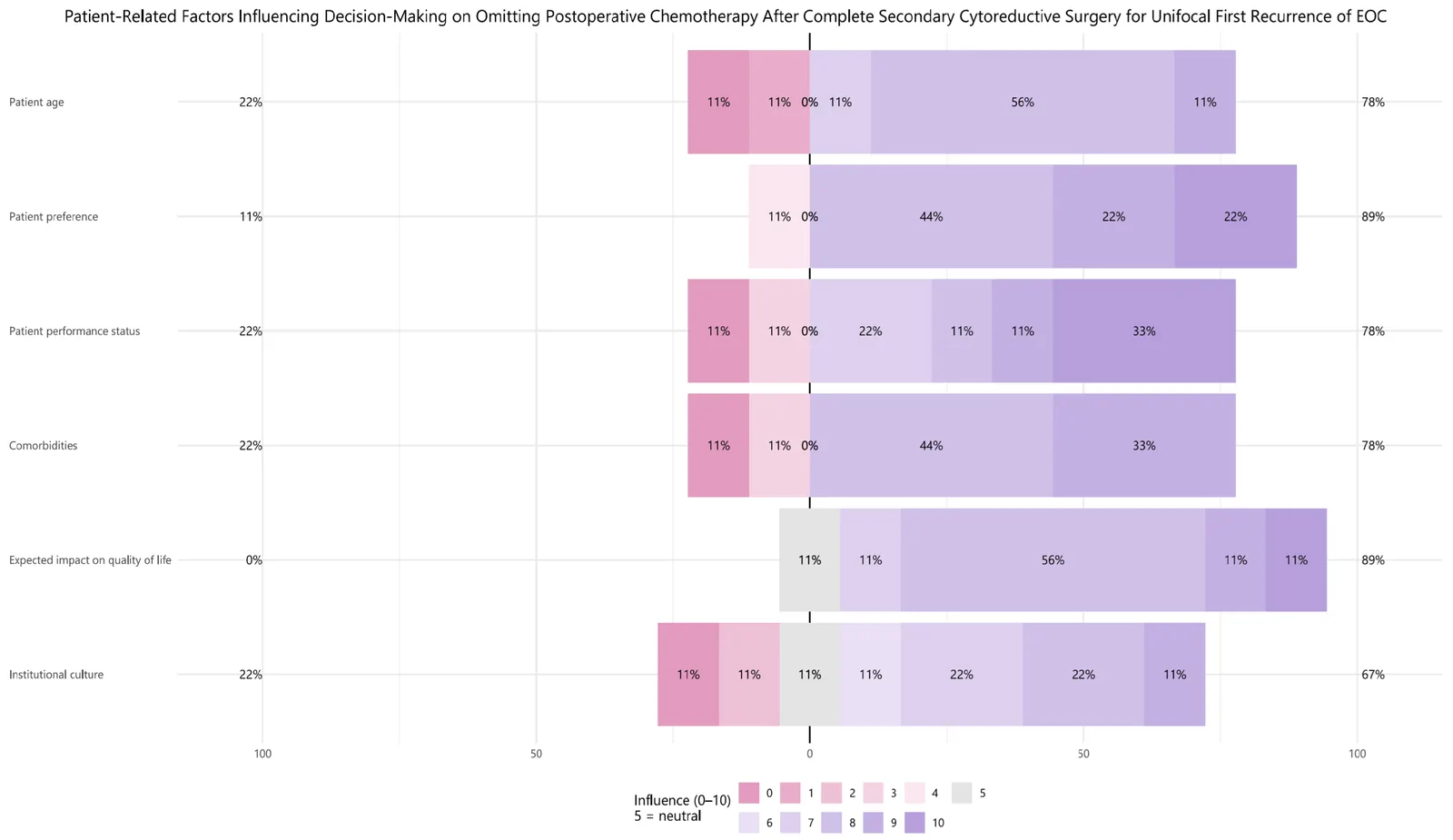
Patient-related factors influencing the decision to omit postoperative chemotherapy after secondary cytoreductive surgery in unifocal first recurrence of EOC (*n* = 9). Ratings of the influence of patient age, patient preference, performance status, comorbidities, the expected impact on quality of life, and institutional culture, each on a 0–10 scale of influence (5 = neutral). Percentages may not sum to 100% due to rounding.

Among the nine respondents who indicated that postoperative chemotherapy was not administered to all patients, a range of clinician- and treatment-related and patient-related factors was rated for their influence on the decision regarding postoperative chemotherapy after complete SCS, each on a 0–10 scale (Figure 2 and Figure 3). Of the clinician- and treatment-related factors, institutional protocol (*n* = 7/9; 78%), disease-free interval (*n* = 6/9; 67%), and toxicity of previous chemotherapy (*n* = 6/9; 67%) were rated most influential, whereas the extent of prior therapy (*n* = 4/9; 44%) was rated lowest. In free-text comments, respondents additionally noted the histologic subtype—adjuvant chemotherapy being given routinely in high-grade serous carcinoma but not in low-grade disease—and the wish to preserve treatment options for subsequent recurrences. Among patient-related factors, patient preference (*n* = 8/9; 89%) and the expected impact on quality of life (*n* = 8/9; 89%) carried the greatest weight, followed by patient age, performance status, and comorbidities (*n* = 7/9; 78% each). Free-text comments here highlighted the patient’s social network and social or psychological factors by one respondent.

### 3.6. Confidence in Recommending Observation and Perceived Barriers

**Figure 4:**
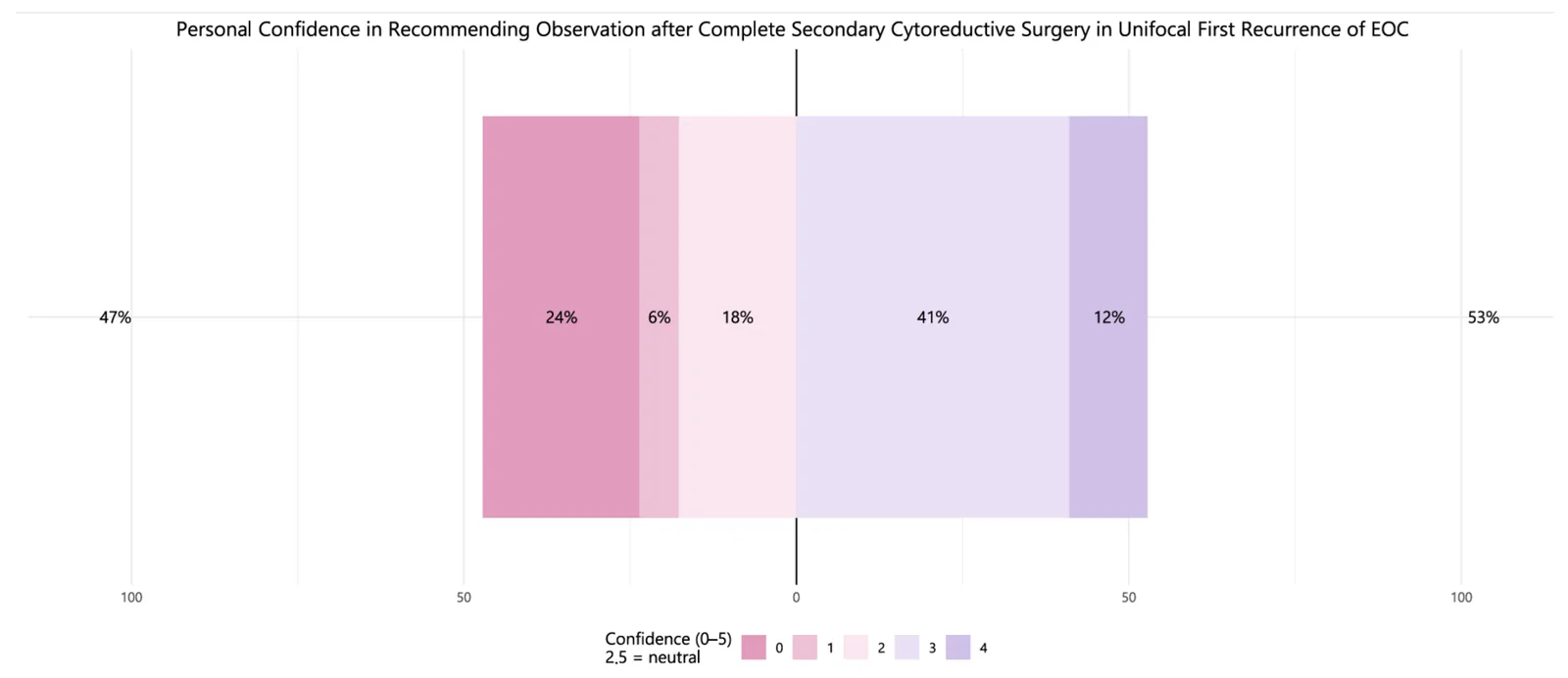
Experts’ views on postoperative chemotherapy after secondary cytoreductive surgery in unifocal first recurrence of EOC (*n* = 17). Ratings of the perceived clinical added value, the strength and clinical relevance of current evidence, and the influence of guidelines on the recommendation of postoperative chemotherapy after secondary cytoreductive surgery in unifocal first recurrence of EOC, each on a 5-point Likert scale (1 = lowest, 3 = moderate, 5 = highest). Unsure responses were excluded. Percentages may not sum to 100% due to rounding.

**Figure 5:**
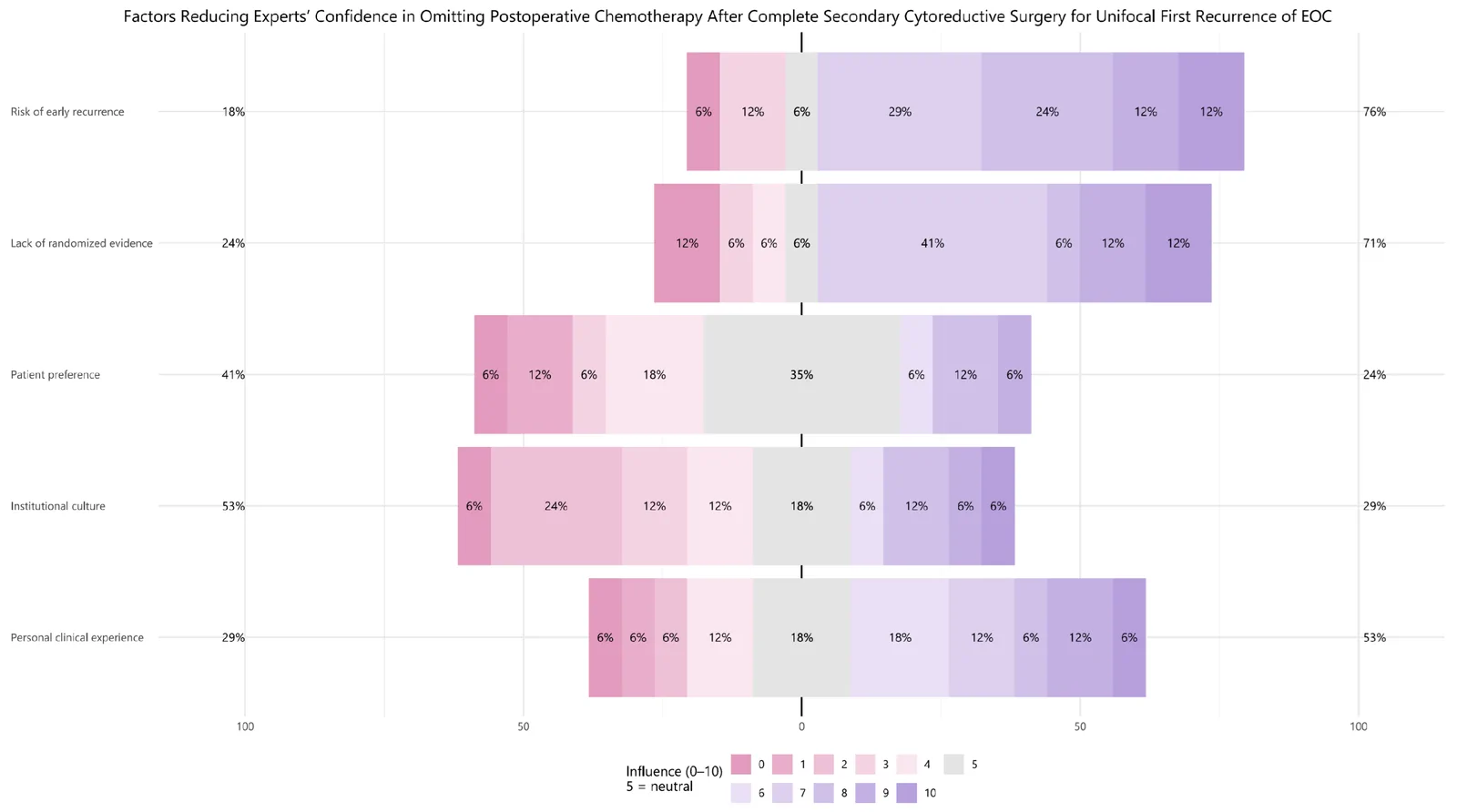
Factors reducing respondents’ confidence in omitting postoperative chemotherapy after secondary cytoreductive surgery in unifocal first recurrence of EOC (*n* = 17). Ratings of the influence of the risk of early recurrence, the lack of randomized evidence, patient preference, institutional culture, and personal clinical experience, each on a 0–10 scale of influence (5 = neutral). Percentages may not sum to 100% due to rounding.

Among the 17 respondents, 53% (*n* = 9/17) felt confident in personally recommending observation alone rather than postoperative chemotherapy, whereas 47% (*n* = 8/17) did not (Figure 4). The factors that most strongly limiting confidence were the risk of early recurrence (*n* = 13/17; 76%) and the lack of randomized evidence (*n* = 12/17; 71%), whereas institutional culture was the least influential (Figure 5). One respondent remarked that, in the absence of symptomatic disease to palliate, chemotherapy should be withheld.

### 3.7. Attitudes Toward a Randomized Trial

A randomized controlled trial comparing observation only with postoperative chemotherapy after complete SCS for a first unifocal recurrence of EOC was considered useful by 14 (*n* = 14/17; 82%) of respondents, and 13 respondents (*n* = 13/14; 76.5%) were willing to participate.

## 4. Discussion

In this survey among gynecological and medical oncologists from international expert oncological centers, we evaluated current clinical practice and factors influencing the decision-making to omit postoperative chemotherapy after complete SCS for a first unifocal recurrence of EOC. A key finding was the absence of a uniform definition of unifocal recurrence. Respondents differed in both the number of lesions and the anatomical locations they considered compatible with unifocal disease. This variation is consistent with our systematic review, in which studies applied different definitions of unifocal recurrence, most commonly one to two lesions, as well as with the broader literature, where terms such as unifocal, isolated, localized, limited, and oligometastatic recurrence are used inconsistently and without a universally accepted definition [10]. Consequently, respondents’ estimates of the annual number of patients undergoing SCS for unifocal recurrence varied according to the definition applied. Under their own local definition, respondents reported treating a median of five patients per year, whereas application of a standardized definition of a single lesion, irrespective of location, reduced this estimate to 3.5 patients per year. A consensus definition is therefore needed before outcomes can be reliably compared across studies and clinical practice.

The clinical uncertainty identified in the literature was also reflected in our survey. Respondents considered postoperative chemotherapy to provide meaningful clinical benefit despite acknowledging that the supporting evidence is limited, and their recommendations were strongly influenced by national and international guidelines. This finding is unsurprising, as direct evidence for this specific clinical scenario is lacking. DESKTOP-III and SOC-1 demonstrated improved outcomes after SCS followed by postoperative chemotherapy but were not designed to evaluate omission of chemotherapy after complete resection of a unifocal recurrence [1,6]. DESKTOP-III did not distinguish between unifocal and multifocal recurrence, whereas SOC-1 included patients with one to three lesions rather than exclusively those with a single lesion. Across both randomized trials, survival benefit was confined to patients achieving complete gross resection, and postoperative chemotherapy was administered to nearly all patients undergoing SCS, with completion rates of 76.7% in DESKTOP-III and 96.7% in SOC-1. Consequently, omission of postoperative chemotherapy after complete SCS has never been prospectively evaluated, and neither trial reported whether chemotherapy administration varied according to resection status or lesion number, consistent with respondents’ perception that the available evidence is weak.

Despite the absence of direct evidence, routine postoperative chemotherapy following SCS, as evaluated in DESKTOP-III, was not uniformly adopted in clinical practice. A proportion of respondents did not routinely administer postoperative chemotherapy and reported managing a median of one patient annually with observation alone following complete SCS. The decision to omit chemotherapy reflected a combination of institutional, clinical, and patient-related factors. Institutional protocols played an important role, alongside clinical considerations including the disease-free interval and previous chemotherapy-related toxicity. Patient preference and the anticipated impact on quality of life were also important determinants. However, DESKTOP-III did not demonstrate clinically meaningful differences in global health-related quality of life between treatment groups [1]. More than half of the 20 respondents reported feeling confident omitting postoperative chemotherapy after complete SCS in patients with a first unifocal recurrence of EOC. Remaining uncertainty among respondents was primarily driven by concerns regarding risk of early recurrence and the lack of randomized evidence.

Taken together, these findings suggest variation in current clinical practice and highlight the lack of evidence to guide decision-making regarding postoperative chemotherapy after complete SCS for a first unifocal recurrence of EOC. This uncertainty was recognized by most respondents, with 14 (82%) respondents considering a randomized trial comparing postoperative chemotherapy with observation only after complete SCS in unifocal first recurrence of EOC to be clinically valuable. Such a trial would require a standardized definition of unifocal recurrence, for instance a single lesion irrespective of location, to ensure a comparable study population. Patients would be randomized to SCS followed by observation only versus SCS followed by postoperative chemotherapy as applied in DESKTOP-III, with progression-free survival and overall survival as primary outcomes. Given the low annual patient volume per center, international collaboration and an adequate sample size would be essential to achieve sufficient inclusions within a feasible timeframe.

### Strengths and Limitations

To our knowledge, this is the first study to assess clinical decision-making regarding the added value of postoperative chemotherapy after SCS in unifocal first recurrent EOC. A major strength is the systematic application of the CFIR 2022 framework to newly develop the questionnaire, ensuring that determinants were captured across the innovation, outer-setting, inner-setting, and individual domains rather than identified ad hoc. This provides a reproducible framework for future implementation-oriented research [15]. This study addresses a rare clinical scenario with small patient numbers per center, which makes international collaboration and knowledge sharing essential to advance evidence on this topic.

This study has several limitations. First, it is subject to bias from several sources. Expert oncological centers were identified from the literature and conferences, introducing selection bias. Not all invited experts responded and three of the completed questionnaires were incomplete, resulting in a response rate of 41.7% and a risk of non-response bias. Responses were also geographically concentrated, with all respondents based in Europe and the majority practicing in the Netherlands. The findings may therefore not capture the full spectrum of international practice, and may in particular reflect Dutch clinical practice more strongly than that of other European or non-European countries. Responses reflect self-reported practice rather than actual treatment data and may therefore be subject to recall bias and may not fully correspond to real-world decision-making. These findings should therefore be interpreted with caution. Second, respondents were asked to apply a single definition of unifocal recurrence, a single lesion irrespective of location, even where this differed from their own practice. This improved comparability but may have further decoupled responses from actual clinical practice. Third, the questionnaire was not formally validated, and the predominantly multiple-choice and slider-based format, while suited to comparison, may have oversimplified nuanced indications and led to a loss of clinically relevant detail.

## 5. Conclusions

Reported practice regarding postoperative chemotherapy after complete SCS for a first unifocal recurrence of EOC varied among participating international experts. The absence of a uniformly applied definition of unifocal recurrence further limits comparison of outcomes across studies and clinical practice, highlighting the need for a shared definition to facilitate data pooling and international collaboration. Given the lack of direct evidence regarding omission of postoperative chemotherapy after complete SCS, an international randomized trial applying a uniform definition of unifocal recurrence, comparing SCS followed by observation with SCS followed by postoperative chemotherapy, is warranted and was considered clinically valuable by most respondents.

## Figures and Tables

**Table 1:** Baseline characteristics of respondents.

Characteristic	Respondents, *n*/*N* (%)
**Country of practice**	
Germany	1/20 (5%)
Italy	1/20 (5%)
The Netherlands	13/20 (65%)
Norway	1/20 (5%)
Spain	1/20 (5%)
Turkey	1/20 (5%)
United Kingdom	2/20 (10%)
**Type of center**	
Secondary referral center	4/20 (20%)
Tertiary referral center	14/20 (70%)
Other	2/20 (10%)
**Primary field of expertise**	
Gynecological oncology	18/20 (90%)
Medical oncology	2/20 (10%)
**Years in practice**	
<5 years	1/20 (5%)
5–10 years	2/20 (10%)
>10 years	17/20 (85%)

**Table 2:** Local definitions of unifocal EOC recurrence reported by respondents.

Definition Criterion	Respondents, *n*/*N* (%)
**Number of lesions accepted as unifocal** (*n* = 19)	
Single lesion only	11/19 (58%)
Up to 2 lesions	1/19 (5%)
Up to 3 lesions	7/19 (37%)
**Anatomical location considered relevant** (*n* = 20)	
Yes	14/20 (70%)
**If relevant, unifocal defined as** (*n* = 14, multiple answers allowed)	
Multiple lesions within a single organ	4/14 (29%)
Multiple lesions within a single anatomical compartment	5/14 (36%)
Multiple lesions within a single lymph node region	6/14 (43%)

## Data Availability

No new data were created or analyzed in this study. Data sharing is not applicable to this article.

## Abbreviations

The following abbreviations are used in this manuscript:

SCS	Secondary cytoreductive surgery
EOC	Epithelial ovarian cancer
PET-CT	Positron Emission Tomography en Computed Tomography
HR	Hazard ratio
CI	Confidence interval
ESGO	European Society of Gynaecological Oncology
ESMO	European Society for Medical Oncology
CFIR	Consolidated Framework for Implementation Research

## Appendix A

Search Strategy

Part 1: (“Ovarian Neoplasms”[MeSH] OR ovarian cancer[tiab] OR epithelial ovarian[tiab] OR ovarian neoplasm*[tiab]) AND Part 2: (recurren*[tiab] OR relapse*[tiab] OR relaps*[tiab] OR “platinum-sensitive”[tiab] OR “platinum sensitive”[tiab]) AND (“Cytoreductive Surgical Procedures”[MeSH] OR cytoreductive surger*[tiab] OR cytoreduction[tiab] OR debulking[tiab] OR “secondary cytoreduct*”[tiab] OR “secondary debulk*”[tiab] OR “secondary surgery”[tiab] OR “repeat cytoreduct*”[tiab] OR “tertiary cytoreduct*”[tiab] OR “secondary cytoreduction”[tiab] OR SCS[tiab])

Filter: 2020–2025, clinical trial, randomized clinical trial

## Appendix B

Questionnaire
I give permission to participate in this survey study.

- Yes
- No

Baseline characteristics
  Q1. What is your country of practice?

Q2. What is the name of the hospital you work in?

Q3. In what type of center do you work?

- Secondary referral center
- Tertiary center
- Other:

Q4. What is your primary field of expertise?

- Gynecological oncology
- Medical oncology

Q5. How many years have you been in practice?

- <5 years
- 5–10 years
- >10 years

Surgical Indication and Definition of Unifocal Recurrence
  Q6. Approximately how many patients with a first unifocal recurrence of epithelial ovarian cancer (EOC) undergo secondary cytoreductive surgery in your center per year?
  Q7. When you refer to an unifocal recurrence of EOC, what is the maximum number of lesions you include under this definition?

- 1 lesion
- 2 lesions
- 3 lesions
- Other (please specify):

Q8. Do you consider the anatomical location relevant when defining unifocal recurrence?

- Yes
- No

If Q8, yes:
  Q8.1. Which of the following would you still consider unifocal recurrence of EOC? (multiple answers allowed):

- Multiple lesions within the same organ (e.g., liver, spleen)
- Multiple lesions confined to an anatomic compartment (e.g., pelvic cavity only)
- Multiple lesions within a single lymph node region (e.g., pelvic, para-aortic, mediastinal)
- Other (please specify)

Remark in questionnaire: For the remainder of this questionnaire, unifocal recurrence of EOC is defined as the presence of a single recurrent lesion, irrespective of anatomical location. We kindly ask you to answer the following questions using this definition, even if it differs from your usual clinical practice.
  Q9. Approximately how many patients with a first unifocal recurrence of EOC (defined as a single lesion) undergo secondary cytoreductive surgery in your center per year?
  Clinical value, Evidence, and Decision-making on Postoperative Chemotherapy after Complete Secondary Cytoreductive Surgery
  Q10. In patients with a first unifocal recurrence who undergo secondary cytoreductive surgery, how would you rate the added clinical value of postoperative chemotherapy?

- No added value
- Limited added value
- Moderate added value
- Significant added value
- Very significant added value
- Unsure

Q11. How would you rate the strength and clinical relevance of the current evidence supporting postoperative chemotherapy in this setting?

- Very weak/insufficient
- Limited
- Moderate
- Strong
- Very strong
- Unsure

Q12. To what extent do national or international guidelines influence your recommendation regarding postoperative chemotherapy after secondary cytoreductive surgery?

- Not at all influential
- Slightly influential
- Moderately influential
- Very influential
- Completely determines my decision
- Unsure

Clinician-Related Factors Influencing Postoperative Chemotherapy Indication after Complete Secondary Cytoreductive Surgery
  Q13. According to the DESKTOP-III trial, all patients with a first unifocal EOC recurrence who undergo complete secondary cytoreductive surgery are treated with postoperative chemotherapy. Is this also the standard approach at your institution?

- Yes, all patients receive postoperative chemotherapy
- Yes, most patients receive postoperative chemotherapy
- No, postoperative chemotherapy is not routinely given
- Unsure/varies by case

If Q13, not answer A:
  Q13.1. To what extent do the following clinical and treatment-related factors influence the decision-making regarding omitting postoperative chemotherapy after complete secondary cytoreductive surgery in patients with a first unifocal EOC recurrence? (One slider per item; 0 = no influence, 10 = very strong influence)

− Disease-free interval (0–10)
− Extent of prior systemic therapy (0–10)
− Toxicity from previous chemotherapy (0–10)
− Lack of high-level evidence (0–10)
− Institutional protocol/local consensus (0–10)
− Opinion of a key specialist (e.g., gynecologic oncologist/medical oncologist) (0–10)
− Other (please specify): (0–10)

Patient-Related Factors Influencing Postoperative Chemotherapy Indication after Complete Secondary Cytoreductive Surgery
  If not Q13, answer A:
  Q13.2 To what extent do the following patient-related factors influence the decision-making regarding omitting postoperative chemotherapy after complete secondary cytoreductive surgery in patients with a first unifocal EOC recurrence? (One slider per item; 0 = no influence, 10 = very strong influence)

− Age (0–10)
− Patient preference (0–10)
− Patient performance status (0–10)
− Comorbidities (0–10)
− Expected impact on quality of life (0–10)
− Institutional culture (0–10)
− Other (please specify): (0–10)

Q14. Approximately how many patients with a first unifocal recurrence of epithelial ovarian cancer (defined as a single lesion) who underwent complete secondary cytoreductive surgery were managed with observation in your center per year (and had NO postoperative chemotherapy)?
  Expert Opinion in Omitting Postoperative Chemotherapy after Complete Secondary Cytoreductive Surgery
  Q15. How confident do you feel personally recommending observation instead of chemotherapy after secondary cytoreductive surgery? (Slider; 0 = Not confident at all, 5 = fully confident)
  If Q15 is smaller than 5:
  Q16. What extent do the following factors reduce your confidence to omit postoperative chemotherapy after complete secondary cytoreductive surgery in patients with a first unifocal EOC recurrence? (One slider per item; 0 = does not limit my confidence, 10 = severely limits my confidence)

− Risk of early recurrence (0–10)
− Lack of randomized evidence (0–10)
− Patient preference (0–10)
− Institutional culture (0–10)
− Personal clinical experience (0–10)
− None/I feel confident omitting chemotherapy (0–10)
− Other (please specify) (0–10)

Feasibility of Randomized Controlled Trials and Research Interest
  Q17. Do you think a randomized controlled trial (RCT) comparing postoperative chemotherapy versus observation after secondary cytoreductive surgery in first unifocal recurrent EOC would be useful?

- Yes
- No

Q18. Would your center be willing to participate in such an RCT?

- Yes
- No
